# Diagnostic value of diffusion-weighted imaging-derived apparent diffusion coefficient and its association with histological prognostic factors in breast cancer

**DOI:** 10.3892/ol.2019.10651

**Published:** 2019-07-22

**Authors:** Congcong Ren, Yu Zou, Xiaodan Zhang, Kui Li

**Affiliations:** Department of Radiology, Women's Hospital, School of Medicine, Zhejiang University, Hangzhou, Zhejiang 310006, P.R. China

**Keywords:** breast cancer, apparent diffusion coefficient, diagnosis, prognosis

## Abstract

Diffusion-weighted imaging (DWI) has been proven to be effective in detecting breast malignancies and has been widely implemented for breast imaging. However, the exact association between certain DWI biomarkers and well-known prognostic factors remains to be fully elucidated. By studying the association between the apparent diffusion coefficient (ADC) and prognostic factors, the present study aimed to explore the diagnostic value and prognostic potential of the ADC in breast lesions. The study included 539 female subjects with histopathologically confirmed breast lesions who underwent DWI of the breast tissue. The diagnoses comprised 307 subjects with malignant breast tumors and 232 with benign breast tumors. The maximum ADC and mean ADC (ADC_mean_) values of the breast lesions were calculated. For malignant tumors, the association between ADC and major prognostic factors, including histological grade, nuclear grade and lymph node status, as well as estrogen receptor (ER), progesterone receptor (PR), human epidermal growth factor receptor 2 (HER-2) and proliferation marker protein Ki-67.(Ki-67) status, were evaluated. The ADC_mean_ demonstrated the best diagnostic performance in distinguishing between malignant and benign lesions. With the optimum cut-off value at 1.30×10^−3^ mm^2^/sec, ADC_mean_ had a sensitivity and specificity of 84.1 and 90.2%, respectively. In those patients with malignant breast lesions, a decreased ADC was associated with breast lesions with high nuclear and histological grades, and lymph node-positive, ER-negative, PR-negative and HER-2-negative status, and Ki-67 ≥14%. In conclusion, the ADC is a useful imaging biomarker for differentiating between benign and malignant breast tumors. The marked association between the ADC and prognostic factors also demonstrated its value in evaluating the malignancy of breast lesions.

## Introduction

Affecting >2.1 million females each year, breast cancer is the primary cause of cancer-associated mortality among females and remains an increasing health threat in developed and developing countries. Despite of this serious situation, the overall survival rate has significantly increased due to screening programs and improved treatment. Various screening and follow-up examinations, including annual mammograms, ultrasounds, computed tomography and magnetic resonance imaging (MRI) scans, are recommended by various guidelines (1). Among them, MRI has demonstrated a relatively increased sensitivity (2) in breast cancer detection compared with mammograms and ultrasounds, which have reported overall sensitivities of 30–60 and 40–80%, respectively (3). In addition, considering the factors of radiation safety and image quality, MRI has its own unique advantages compared with mammography. However, conventional MRI technologies also have limitations, including relatively low specificity (2), which may lead to difficulties in distinguishing malignant tumors from benign ones, and are therefore unable to determine overall prognosis; as a result, a proportion of patients undergo unnecessary biopsies. In comparison, diffusion-weighted imaging (DWI) and its derived measurements, including the apparent diffusion coefficient (ADC), allow for improved characterization of the biological properties of tissues, and this technique has therefore been recognized for its superior oncology applications.

Different from conventional dynamic contrast-enhanced (DCE) MRI, DWI requires no administration of contrast agents and quantifies tissue cellularity by measuring the Brownian motion of water molecules (4). Previous studies have repeatedly demonstrated decreased water diffusion, which appears brighter on DWI (5,6) and darker on ADC maps, and higher cellularity in malignant breast lesions compared with normal fibroglandular tissue. ADC has also been widely used to distinguish malignant breast cancer from benign lesions. Min *et al* (7) revealed that benign lesions exhibited an increased mean ADC value compared with their malignant counterparts regardless of b-values. They also demonstrated that DWI achieved a sensitivity of 82.8% and specificity of 90%, with a cutoff ADC value of 1.23×10^−3^ mm^2^/sec. With a promising differential diagnostic value in clinical practice, DWI and ADC are now integrated into routine MRI breast examination protocols, primarily to distinguish between benign and malignant lesions (8,9). However, only a limited number of studies have examined the association between ADC and clinical prognostic factors.

Traditional prognostic factors for breast cancer include histology, staging (size and axillary node involvement), tumor grading, heredity, obesity, smoking and molecular markers (10). Among them, molecular markers including estrogen receptor (ER), progesterone receptor (PR) and human epidermal growth factor 2 receptor (HER-2) status are currently widely used as indicators to guide adjuvant therapy and predict long-term outcomes (11). As MRI examinations are less expensive and more available in developing countries compared with Oncotype DX, the development of MRI-derived biomarkers for breast cancer has been a focus of numerous studies. Although the potential of ADC in assessing malignancy of breast lesions was demonstrated by previous studies (6,12,13) and the ADC exhibited a correlation with certain prognostic factors, these results have not been verified in large Chinese populations. Therefore, considering the fact that pre-operative evaluation of the extent and prognosis of breast cancer is critical to clinical practice, its optimization may consequently improve the overall survival rate and outcomes of patients with breast cancer. The aims of the present study were as follows: i) Investigate the diagnostic performance of DWI-derived ADC values in differentiating malignant tumors from benign ones; and ii) examine the correlation between ADC values and various prognostic factors in females with breast cancer.

## Materials and methods

### 

#### Study cohort

The present cross-sectional study was performed at the Department of Radiology of the Women's Hospital, School of Medicine, Zhejiang University (Hangzhou, China) from November 2015 to September 2018. DWI was included in the clinical breast MRI protocol for females with suspicious breast lesions detected by mammogram and/or sonography. The study protocol was approved by the Institutional Review Board and Ethics Committee of Women's Hospital, School of Medicine, Zhejiang University. Written informed consent was obtained from all the subjects prior to the study.

The present study retrospectively assessed 762 female patients with breast masses who had undergone DWI prior to histopathological diagnosis from specimens obtained via core needle biopsy or excision surgery. Patients with inflammatory cancer or those receiving ongoing chemotherapy, and those without histopathological confirmation of the lesion, were excluded. Patients with tumorous lesions >10 mm in diameter were excluded to avoid unreliable delineation of the tumor on MRI. Based on these criteria, 223 patients were excluded and 539 patients were eventually included in the present study.

#### Histological analysis

Histological analysis was performed on tissues obtained by core needle biopsy and excision surgery. Histological grading of breast lesions was performed considering three morphological features: Tubule formation, nuclear pleomorphism and the number of mitotic figures, according to the criteria of Elston and Ellis (14). The total possible score ranged from 3–9, with a total score of 3–5 representing grade 1, a total score of 6 or 7 resembling grade 2 and a total score of 8 or 9 resembling grade 3. The nuclear grade (1, differentiated; 2, moderately differentiated; and 3, poorly differentiated) was determined from 10% formalin-fixed paraffin-embedded tumor tissue sections stained with hematoxylin and eosin based on Robinson's grading system (15). Immunohistochemistry (IHC) was performed on tissues fixed with 10% neutral buffered formalin at room temperature for 18 h. Paraffin-embedded materials were cut to 5-µm sections (16,17), deparaffinized in xylene, treated with 100% ethanol at room temperature and then heated in a microwave in 0.01 M citrate buffer (pH 6.0) for 15 min for antigen retrieval. ER (ER: Era EP1), PR (PR: PgR, 636) and HER-2 (c-erbB-2) status, and proliferation index, determined using proliferation marker protein Ki-67 (DAKO; Agilent Technologies, Inc.; Ki-67, MM-1) (1:100) antibody, were examined on all specimens using a DAKO Autostainer (Dako; Agilent Technologies, Inc.) and commercially available monoclonal antibodies. The proportion of ER-positive and PR-positive tumor cells was expressed as a percentage. ER and PR expression were scored as positive or negative with a nuclear immunostaining cut-off of 10%. For ER/PR status to be considered positive, guidelines from the American Society of Clinical Oncology and College of American Pathologists (18) recommended that ≥1% of tumor cells must demonstrate positive nuclear staining on IHC. However, in routine practice, a wide range of arbitrary cutoffs in proportion of stained cells are used. For example, 1 (19), 5–10, and 20% (20). A number of clinicians (21–23), including our hospital, consider 10% as the cut-off for eligibility for endocrine therapy. In addition, Iwamoto *et al* (24) demonstrated that tumors with <10% ER-positive staining on IHC have molecular characteristics more similar to the ER-negative, basal-like phenotype. Considering the fact that patients with ER-positive (1–9%) tumors do not appear to benefit from endocrine therapy (25), a cut-off at 10% was adopted in the present study. HER-2 expression was evaluated as positive when membrane immunostaining scores were 3+, or when HER-2 gene amplification was demonstrated by fluorescent *in situ* hybridization in case of a sample with 2+ score, based on the scoring guidelines of HercepTest (DAKO; Agilent Technologies, Inc.) (26). Ki-67 staining results were expressed as the percentage of Ki-67-positive malignant cells among 1,000 malignant cells assessed under high-power magnification (×40 objective). Samples with ≥14% Ki-67 staining were considered positive, while those with <14% were considered negative according to the St. Gallen consensus (27). Information on the microvascular lymph node status was obtained by sentinel lymph node resection followed by immediate lymph node dissection. A positive result was defined as presence of metastasis. The prognostic markers considered in the present study were histological and nuclear grade, lymph node status and molecular markers, including ER, PR, HER-2 and Ki-67.

#### MRI acquisition and analysis

Diagnostic MRI was performed on a GE Signa HDX 1.5 T MRI machine (GE Healthcare) using a double breast coil with the patient in a prone position. For all cases, a standardized MRI protocol was applied.

Prior to contrast medium administration, axial fat-suppressed T2-weighted short-tau inversion recovery images [response time (TR), 5,220 msec; echo time (TE), 49.2 msec; inversion time (TI), 145 msec; field of view (FOV), 36×36 cm; slice thickness, 5.5 mm; total acquisition time, 2 min 32 sec] were obtained. Axial DWI with spin-echo planar imaging was performed with the following parameters: b=1,000 sec/mm^2^; TR, 4,250 msec; TE, 76.5 msec; FOV, 39×27.3 cm; slice thickness, 5.5 mm; acquisition time, 60 sec.

In all patients, a bolus of intravenous contrast medium (gadopentetate dimeglumine) was administered at a dose of 0.2 mmol/kg body weight (0.5 mmol/ml Magnevist^®^), followed by 10 ml saline solution (1%). Dynamic MRI (VIBRANT^®^) with fat suppression was performed prior to and 6 times after injection of the contrast medium. The parameters for dynamic MRI were as follows: TR, 5.7 msec; TE, 2.8 msec; TI, 18 msec; FOV, 37×33.3 cm; slice thickness, 1.6 mm; acquisition time, 6 min 14 sec.

The ADC map was automatically generated by the console of the manufacturer (AW VolumeShare 5; GE Healthcare). Linear regression was used to calculate ADC maps. Breast lesions were reviewed and manually delineated by 2 independent radiologists with 10 and 5 years' experience in breast MRI, respectively, and blinded to other imaging or clinicopathological findings other than the presence of breast masses. The lesions were manually delineated with a circular region of interest placed within the primary lesions to include an area as large as possible within the confines of the actual lesions. The mean and maximum ADC values of lesions were denoted as ADC_mean_ and ADC_max_, respectively. ADC measurements were performed at least 3 times by 2 independent observers and the average ADC was recorded as the final result. The volume of the lesions was calculated by counting the number of voxels delineated on lesion maps and then multiplied by the size of the voxel.

#### Statistical analysis

For continuous variables, the Kolmogorov-Smirnov test was performed. For comparisons between breast cancer and different types of benign lesion, a Student's t-test was used. In addition, a receiver operating characteristics (ROC) curve was fitted and the area under the ROC curve (AUC) with 95% confidence interval (CI) was determined to identify the best cut-off ADC value for differentiating between benign and malignant breast masses. Sensitivity, specificity, positive predictive values and negative predictive values were calculated, respectively.

The associations between ADC values and prognostic factors were calculated using Student's t-test with Bonferroni correction. The ADC values of histological grade (1 vs. 2/3), nuclear grade (1 vs. 2/3), lymph node status (positive vs. negative), as well as status of ER, PR, HER-2 (positive vs. negative) and Ki-67 (<14 vs. ≥14%) were compared. In the univariate analysis, variables for which P<0.1, including histological and nuclear grade, lymph node status, ER, PR, HER-2 and Ki-67, were subjected to multiple logistic regression analysis to determine those that were independently associated with ADC_max_ and ADC_mean_ values. P<0.05 was considered to indicate a statistically significant difference. SPSS 22.0 (IBM Corp.) was used for all statistical analyses.

## Results

**Figure 1.**
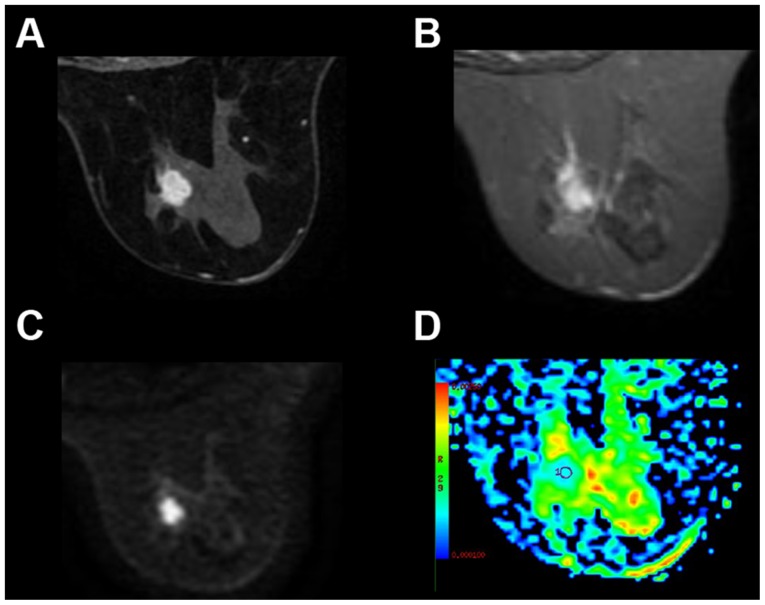
Representative imaging results from a 51-year-old patient with infiltrating ductal carcinoma in the right breast. The tumor was histological grade 2, without axillary lymph node metastasis. Immunohistochemical staining exhibited positive estrogen receptor and progesterone receptor expression, negative human epidermal growth factor receptor 2 expression and a proliferation index (proliferation marker protein Ki-67) of 30%. (A) Dynamic early enhancement image showing an irregular, heterogeneously enhancing lesion with a lobulated margin. (B) The maximum diameter of the tumor was 1.5 cm on T2WI imaging results. (C) Diffusion weighted imaging results demonstrated a high signal intensity lesion in the right breast tissue. (D) The ADC map revealed increased diffusion (ADC_mean_=0.74×10^−3^ mm^2^/sec) within the index lesion. ADC, apparent diffusion coefficient.

**Figure 2.**
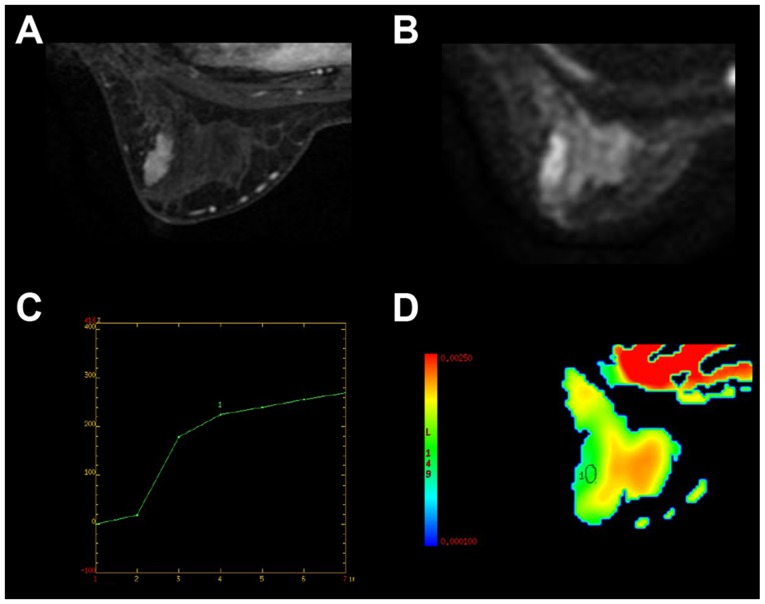
Representative imaging results from a 35-year-old patient with fibroadenoma in the left breast. (A) The maximum diameter of the tumor was 2.6 cm, and demonstrated a well-defined, oval heterogeneously enhancing lesion on dynamic enhancement image. (B) The lesion was hyperintense on diffusion weighted imaging scans. (C) Time signal intensity analysis demonstrated a gradual progressive enhancement pattern (type 1 curve). (D) The lesion was hypointense on the ADC map with ADC_mean_=1.26×10^−3^ mm^2^/sec. ADC, apparent diffusion coefficient.

A total of 539 females (mean age, 43.9±8.3 years) with breast lesions were included in the present study. Based on the pathological results, malignant tumors were detected in 307 subjects (Fig. 1) and benign tumors in 232 subjects (Fig. 2). Detailed demographics of the patients and pathological types of their lesions are summarized in Table I. Patients with malignant breast lesions were significantly older compared with those with benign lesions (P<0.001) and had a lower body weight (P=0.007).

**Figure 3.**
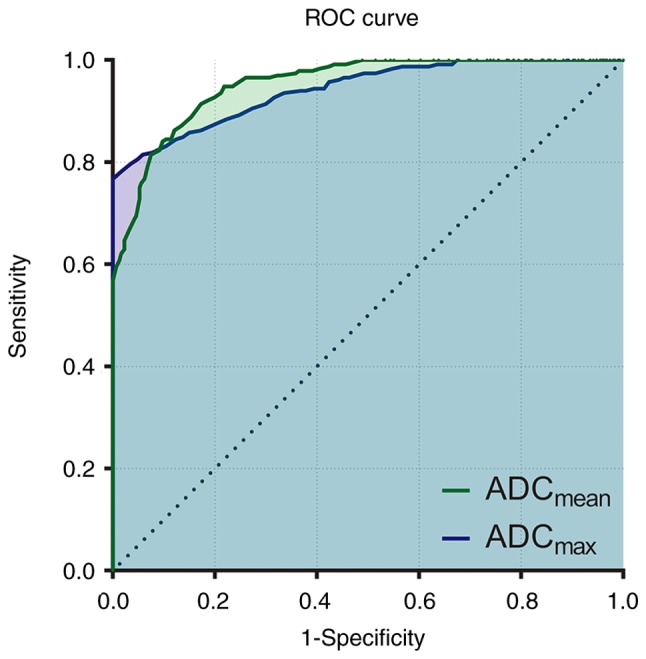
ROC curve for ADC_mean_ and ADC_max_ in differentiation between benign and malignant breast lesions. ROC, Receiver operating characteristic; ADC, apparent diffusion coefficient.

Analysis of the ADC_mean_ and ADC_max_ values of malignant and benign breast lesions indicated that malignant lesions usually had a smaller size (P<0.001) and lower ADC_max_ (P<0.001)/ADC_mean_ (P<0.001) compared with benign lesions (Table I). The ADC_mean_ demonstrated a relatively improved performance in distinguishing malignant from benign lesions compared with ADC_max_, as indicated by the ROC curves in Fig. 3. The AUC for ADC_mean_ and ADC_max_ was 0.953 and 0.942, respectively. With the optimum cut-off for the ADC_mean_ value at 1.30×10^−3^ mm^2^/sec, DWI achieved a sensitivity of 84.1% and a specificity of 90.2%, a positive predictive value of 86.7% and a negative predictive value of 88.2% for malignant vs. benign lesions. Furthermore, with the optimum cut-off value at 1.98 mm^2^/sec, ADC_max_ yielded a sensitivity of 78.4%, a specificity of 98.0%, positive predictive value of 85.8% and negative predictive value of 96.8% regarding the identification of malignant vs. benign lesions.

Next, the association between ADC and independent tumor prognostic factors was examined. Tumor prognostic factors included histological grade, nuclear grade, lymph node status and molecular markers, including ER, PR, HER-2 and Ki-67. The analysis revealed significant associations between ADC and all prognostic factors. For those patients with malignant breast lesions, univariate analysis demonstrated significantly lower ADC_mean_ and ADC_max_ values for high histological grade (grade 2 and 3), high nuclear grade (grade 2 and 3), and lymph node-positive, ER-negative, PR-negative, HER-2-negative status, and Ki-67 ≥14%. Detailed ADC_mean_ and ADC_max_ values for patients with different prognostic factors are presented in Table II.

The present study also performed multiple regression analyses regarding the relative association between prognostic factors for patients with malignant breast lesions and MRI biomarkers (ADC_max_ and ADC_mean_). Histological grade (P<0.001), nuclear grade (P<0.001), Ki-67 (P<0.001) and PR (P=0.005) were the variables demonstrated to independently affect the ADC_max_. Furthermore, Ki-67 (P<0.001), lymph node status (P<0.001), HER-2 (P=0.01) and PR (P=0.01) were all indicated to independently affect the ADC_mean_.

## Discussion

To the best of our knowledge, the present study is the first large-scale study performed in a Chinese population to validate the use of the ADC for distinguishing malignant from benign breast lesions, and to determine its association with common biomarkers of breast malignancy and prognosis. The results demonstrated that ADC is a promising tool in differentiating malignant tumors from benign masses with high sensitivity and specificity, with optional cutoff values of 1.30×10^−3^ and 1.98 mm^2^/sec for ADC_mean_ and ADC_max_, respectively. Furthermore, ADC biomarkers have potential in predicting the clinical outcomes of malignant breast cancer. The present results suggested that ADC_max_ and ADC mean were significantly decreased in patients with high nuclear grade, and lymph node-positive, ER-negative, PR-negative and HER-2-negative status, and Ki-67 ≥14%. Subsequent analysis of the effects of independent prognostic factors on ADC biomarkers suggested that ADC_max_ was affected by nuclear grade, Ki-67 and PR, whereas ADC_mean_ was affected by lymph node status, HER-2, PR and Ki-67.

Breast cancer is one of the most common types of cancer in females worldwide and DCE-MRI is an established technique for detection, diagnosis and staging of breast cancer. Despite an inherently high sensitivity, DCE only has moderate specificity for the characterization of breast lesions (14). In clinical practice, a standardized imaging protocol allows for the investigation of morphological and kinetic patterns of benign and malignant breast lesions screened by mammography and ultrasound. However, this standardized protocol is also prone to a high false-positive rate and may therefore result in unnecessary biopsies. Conversely, being able to measure the biophysical characteristics of tissues, DWI and its derived ADC maps were mostly applied in breast imaging to decrease false-positives on conventional DCE-MRI. Numerous studies have demonstrated that malignant breast lesions usually exhibit a high signal on DWI and a low signal on ADC maps compared with normal fibroglandular tissues (15), indicating decreased water diffusion in malignant tissues. Significantly increased cell density was the major contributor to this decreased water diffusion, consequently resulting in an increased hindrance of water motion in the tortuous extracellular space and increased volume of restricted intracellular fluid (16,17). For example, simple cysts demonstrate higher water diffusion rates compared with breast fibroglandular tissue due to the relatively unrestricted microenvironment. The potential application of DWI and ADC may avoid unnecessary biopsies and provides a fast approach to assess the malignancy of suspicious breast lesions (18). A meta-analysis of 14 studies revealed that ADCs demonstrated an excellent performance in classifying suspicious breast lesions, and their inclusion may therefore increase the accuracy of conventional clinical breast assessments (19). ADC_max_ and ADC_mean_ values demonstrated a good performance in the differentiation between malignant and benign breast masses. The ADC_mean_ for malignant and benign tumors in the present study were 1.0±0.2 and 1.5±0.2, respectively. This was, to a certain degree, consistent with the results of previous studies on breast lesions (9,17,20,21). Compared with previous studies, the present study included a large population and yielded more comprehensive results. The ADC_mean_ alone had the best performance in differentiating between benign and malignant diseases, and with the best cut-off value at 1.98 mm^2^/sec, it had a sensitivity of 84.1%, specificity of 90.2%, positive predictive value of 86.7% and negative predictive value of 88.2%. While the present study did not compare the accuracy of DCE with DWI for breast cancer detection, a previous meta-analysis obtained a pooled sensitivity and specificity of DCE-MRI of 93.2 and 71.1% (19). This suggested that DWI alone had an improved specificity compared with DCE-MRI; therefore, this technique may be implemented to complement conventional parameters and improve the accuracy of breast cancer detection. As the measurement of the extracellular water content provides information on additional features, this may increase the specificity of the classification of pathological breast lesions. More importantly, although a generalizable ADC threshold should always be avoided due to dependence of lesion selection and combination of b-values (22), the sensitivity and specificity of DWI were robust and not significantly affected by choice of b-values as long as a suggested maximum b-value of 1,000 sec/mm^2^ was adopted (23). Despite the fact that DWI is not to be used as a stand-alone diagnostic criterion, it is useful if integrated into the conventional clinical protocol.

Considering that personalized and targeted therapeutic approaches largely depend on the accuracy of tumor characterization in terms of histological type and biological aggressiveness, a non-invasive method capable of measuring all prognostic features is more favorable compared with other techniques. From the results of the present study, which demonstrated that malignant breast lesions had lower ADCs, it may be expected that lower ADCs, likely due to higher levels of proliferation and cellularity, would generally correlate with higher aggressiveness. Cellularity is an important indicator of tumor grade. As increased cellular density of high-grade tumors is associated with smaller extracellular volume fractions, tumor cellularity is inversely correlated with tumor ADC. Indeed, Jiang *et al* (28) demonstrated a marked negative correlation between cellularity and ADC. However, the exact association between restricted diffusion and well-known prognostic factors has remained largely unknown. For example, a number of undifferentiated breast tumors have very few neoplastic cells in an abundant fibrous stroma, and this may result in increased ADC values. In certain cases, the complex microarchitecture and strong desmoplasia in tumors makes the reliable detection of disease and early diagnosis difficult. In addition, although fibrous stroma with higher stroma grades (3–5) demonstrate low ADCs just like neoplastic cells (29), the microstructural models for diffusion MRI are always too simplistic to describe the underlying tissue microstructure in clinical settings. This issue was also raised by a previous study (30), and efforts to build more complex models to better describe the data are being made. Therefore, at present, histological assessments remain irreplaceable, and clinical decisions should be made based on the results of multiple tests, to compensate for the limitations of any single treatment modality. In the present study, the correlation between the ADC values and various prognostic factors was assessed. The results suggested that patients with detected lymph node metastasis had a significantly decreased ADC value in the primary breast tumor. This is in concordance with several previous studies (6,25), and it is commonly accepted that a lower ADC is an indicator of higher aggressiveness and metastatic potential. Furthermore, although the prognosis of malignant tumors does not exclusively depend on the histological type of cancer cells, the histopathological characteristics of the tumor, particularly tumor grade, are markedly correlated with tumor progression. The results of the present study demonstrated a statistically significant association between higher nuclear grade and lower ADCs. This is in accordance with other studies (6,26), which all observed increased cellular density in high-grade tumors by measuring ADCs. Furthermore, previous studies have investigated the association between histological grade and ADC (31,32). The results of the present study were in concordance with those identified previously (29,30), and demonstrated an association between lower ADC values and higher histological grades. This subsequently supports the hypothesis that the increased cellular density observed in high-grade malignancies is associated with low ADC values (33). Overexpression of HER-2 accelerates cell growth, thereby contributing to carcinogenesis. Consequently, HER-2-positive cells exhibit a more malignant phenotype compared HER-2-negative cells, comprising increased cell proliferation, invasion and metastatic potential. However, the present study determined increased ADC values in HER-2-positive samples compared with HER-2-negative breast cancer samples. Notably, HER-2 also induces angiogenesis, which leads to increased perfusion in tumors. Based on this, the increased ADC values observed in HER-2-positive lesions in the present study may be explained by an increased proportion of total extracellular fluid due to high vascularity. Although to the best of our knowledge, there is no study investigating the association between vascularity of breast lesions and HER-2 expression, different imaging studies indirectly supported our hypothesis. Zhang *et al* (34) and Rashmi *et al* (35) demonstrated that tumors with high Adler degrees of vascularity were associated with positive HER-2 expression in power doppler studies. Due to the lack of pathological evidence available at present, above statement is just speculative, and histological examination is always required to verify this high vascularity hypothesis. Martincich *et al* (36) also observed high ADCs in tumors with high HER-2 expression compared with tumors without HER-2 expression. Of note, although ER-positive breast cancer generally has an improved prognosis compared with ER-negative cancer, previous studies have described conflicting results, as several studies indicated that ER-positive breast lesions exhibited lower ADCs (12,37,38). The results of the present study indicated increased ADCs in ER-positive lesions, which is only consistent with the study by Kitajima *et al* (39). Similarly, while most studies did not observe any significant association between PR status and ADC, the data from the present study indicated that PR-positive lesions exhibited increased ADC values. Therefore, the correlation of ADC with other prognostic factors, including ER, PR, HER-2 and Ki-67, is less consistent among studies and may vary between different populations. Future large-population studies may be required to determine the association between DWI biomarkers and these prognostic factors.

The present study had several limitations. Firstly, the present study had a retrospective design, was performed at a single institution, and it was not possible to evaluate the long-term follow-up results of the study population. The association between MRI imaging biomarkers and treatment response may be an important topic for future study. Secondly, the present study did not compare the diagnostic performance of the ADC with conventional DCE-MRI. Although the combination of 2 methods provides an improved diagnostic value compared with either alone, it may be worthwhile to assess this specifically in future studies. Finally, all of the patients included in the present study were screened by mammography and/or sonography and had a tumor size of >10 mm, which may have potentially yielded selection bias. One possible consequence of this selection bias may be the increased prevalence of ER-negative, PR-negative and HER-2-postive breast lesions in the present study compared to others. Therefore, the data should be interpreted the with caution and direct comparisons between different studies should be avoided.

In conclusion, the present study revealed that malignant tissues exhibited lower ADCs compared with benign tumors. This suggested that ADCs may be promising imaging parameters that may help identify tumors with higher malignancies. In addition, the present study provided additional confirmatory evidence for the utility of the ADC values in the characterization of breast lesions, and the ADC_mean_ and ADC_max_ were effective parameters to distinguish malignant from benign breast lesions. Therefore, DWI-derived ADCs are useful biomarkers that may contribute to improved concordance between radiological and pathological data.

## Figures and Tables

**Table I.** Patient demographics, histopathological diagnosis and imaging biomarkers for patients with benign and malignant breast lesions (n=539).

Characteristics	Benign (n=232)	Malignant (n=307)	P value
Age, years	41.3±7.5	45.7±7.7	<0.001
Weight, kg	62.2±10.4	59.5±9.8	0.007
Height, cm	158.2±5.3	159.1±5.7	0.352
Final diagnosis, n (%)			
Fibrocystic changes	12 (5.2)	–	–
Plasma cell mastitis	49 (21.0)	–	–
Intraductal papilloma	13 (5.5)	–	
Fibroadenoma	77 (33.3)	–	
Mastopathy	58 (25.1)	–	
Sclerosing mastopathy	23 (10.0)	–	
Infiltrating ductal carcinoma	–	176 (57.3)	
Malignant phyllodes tumor	–	10 (3.2)	
Mucinous carcinoma	–	6 (2.0)	
Invasive lobular carcinoma	–	115 (37.5)	
Volume, ml	7.7±4.1	5.8±3.9	<0.001
ADC_max_, ×10^−3^ mm^2^/s	2.1±0.2	1.7±0.2	<0.001
ADC_mean_, ×10^−3^ mm^2^/s	1.5±0.2	1.0±0.2	<0.001

ADC, apparent diffusion coefficient.

**Table II.** Associations between prognostic factors and ADC measurements for patients with malignant breast lesions (n=307).

		ADC_max_, ×10^−3^ mm^2^/s		ADC_mean_, ×10^−3^ mm^2^/s	
			
Prognostic factors	No. of cases	Mean ± SD	P value	Mean ± SD	P value
Histological Grade			<0.001		<0.001
1	153	1.79±0.12		1.20±0.12	
2+3	154	1.48±0.11		0.83±0.11	
Nuclear grade			<0.001		<0.001
1	160	1.85±0.09		1.18±0.12	
2+3	147	1.56±0.11		0.87±0.08	
Lymph node status			<0.001		<0.001
Positive	148	1.56±0.11		0.88±0.09	
Negative	159	1.85±0.09		1.17±0.12	
ER			<0.001		<0.001
Positive	91	1.80±0.15		1.14±0.17	
Negative	216	1.67±0.17		0.99±0.17	
PR			0.006		<0.001
Positive	67	1.76±0.18		1.13±0.21	
Negative	240	1.69±0.17		1.00±0.17	
HER-2			<0.001		<0.001
Positive	178	1.82±0.12		1.14±0.15	
Negative	129	1.56±0.12		0.88±0.09	
Ki-67			<0.001		<0.001
<14%	103	1.89±0.08		1.24±0.10	
≥14%	204	1.62±0.13		0.92±0.11	

ADC, apparent diffusion coefficient; SD, standard deviation; ER, estrogen receptor; PR, progesterone receptor; HER-2, human epidermal growth factor 2; Ki-67, proliferation marker protein Ki-67.

## Data Availability

All data are available upon reasonable request.
